# Separation of Low-Molecular-Weight Organics by Water-Soluble Macrocyclic Arenes

**DOI:** 10.3390/molecules27238554

**Published:** 2022-12-05

**Authors:** Wenhui Wang, Zheng Li, Chunli Song, Jie Yang, Yingwei Yang

**Affiliations:** International Joint Research Laboratory of Nano-Micro Architecture Chemistry, College of Chemistry, Jilin University, 2699 Qianjin Street, Changchun 130012, China

**Keywords:** low-molecular-weight organics, liquid–liquid extraction, host-guest chemistry, pillararene, separation

## Abstract

In this study, we fabricate a series of water-soluble anionic macrocyclic arenes, including pillar[5]arene (WP5), pillar[6]arene (WP6), leaning pillar[6]arene (WLT6), and biphenyl-extended pillar[6]arene (WBpP6), which show different separation capabilities toward low-molecular-weight organics, such as short chain haloalkanes, cyclic aliphatics, and aromatics, in water. The liquid–liquid distribution experiments are carried out at room temperature. The separation factor for low-molecular-weight organics is evaluated in the extraction of equimolar mixtures. WP6 demonstrates a high extraction efficiency of up to 89% in separating toluene/methylcyclohexane mixtures. These adsorbents also have the advantages of rapid adsorption, high separation efficiency, remarkable selectivity, and good recyclability. This work not only expands the application scope of macrocyclic chemistry, but also has practical research value for organics separation and water purification.

## 1. Introduction

The separation of high-value-added chemical feedstocks using feasible and environmentally friendly methods is always an essential topic in the chemical industry. As common precursors or solvents in the chemical industry, low-molecular-weight organics, such as short-chain haloalkanes, cyclic aliphatics, and aromatics, are widely used in synthetic fibers [1], surfactants [2], varnishes [3], pesticides [4,5], and so on. In particular, the separation of these organics based on their different boiling points is generally realized by conventional distillation. However, many aromatic isomers [6,7], short-chain alkane homologs [8], and cyclic aliphatics [9,10] have similar physical properties and close boiling points, making it difficult to guarantee chemical separation purity. In addition, the utilization of entrainers would cause additional energy and expense waste [11]. Therefore, it is necessary to develop cheap, convenient, and efficient separation methods to meet the requirements of industrial production. Meanwhile, the process of adsorptive separation is considered to be an effective, energy-efficient, and feasible method for separating liquid mixtures with close boiling points [12]. Adsorbents like activated carbon [13,14], zeolite [15], ordered mesoporous silica [16], and supramolecular macrocycles [17,18] have been extensively used to remove pollutants from water. Among them, supramolecular macrocycles, with their high selectivity and high recovery efficiency, emerge as one of the most effective receptors for these low-molecular-weight organics, and may eventually replace the current isolation techniques used to separate these mixtures.

Macrocycles like cyclodextrins [19], calixarenes [20,21], and pillararenes [22,23,24,25] are known for their unique cavity structures, which can accommodate specific guest molecules, as well as their diverse modifications and self-assembly behaviors. Their size-specific electron-rich hydrophobic cavities can selectively bind certain molecules through host–guest interactions [26,27,28]. The host–guest properties of macrocycles have been extensively applied in the fields of drug delivery [29,30,31], water purification [32], self-assembly [33], photochromism [34,35], and substance detection [36,37,38,39]. In particular, the development of pillararene-based macrocyclic arenes has increased rapidly since they were first reported on in 2008, and a variety of expanded versions of macrocyclic arenes, such as leaning pillar[6]arene [40], biphenyl-extended pillar[6]arene [41], geminiarenes [42], leggero pillar[5]arene [43], and hybrid [n] arenes [44], have been synthesized. They have also been widely employed in catalytic processes [45], molecular devices [46], substance detection [47], stimuli-responsive materials [48], medicine, and theranostics [49,50].

**Scheme 1:**
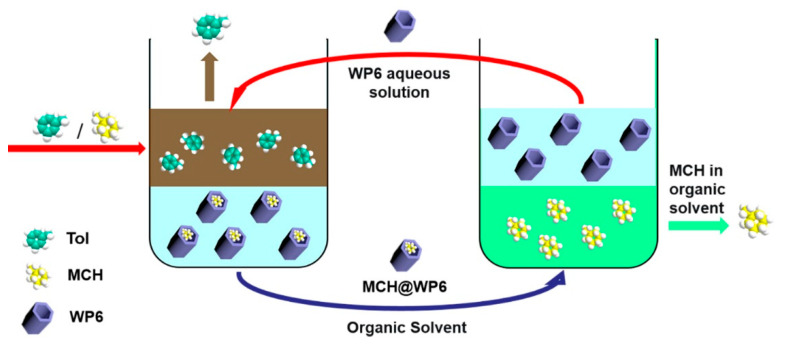
Schematic illustration of the separation process by WP6.

In this work, we successfully synthesized four carboxyl-modified anionic macrocyclic arenes and utilized their water solubility and host–guest properties to separate small organics in the water phase (Scheme 1). Control experiments confirmed that WP6 performed the best when extracting methylcyclohexane (MCH) from toluene (Tol) mixtures. This is the first report of a pillar[6]arene aqueous solution being applied in an extraction. Investigating the performance of water-soluble pillararenes as adsorptive separation materials for low-molecular-weight organics as we have done can provide a lot of valuable information.

## 2. Results

**Figure 1:**
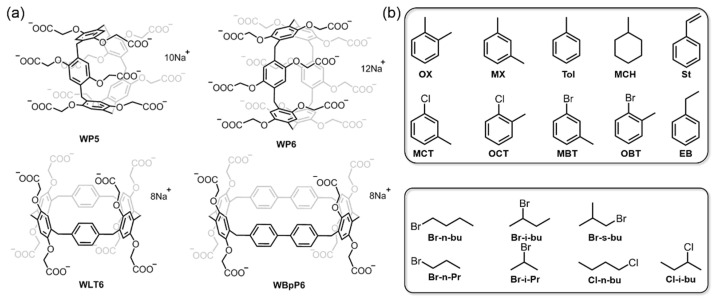
Chemical structures of (**a**) four water-soluble anionic macrocyclic arenes and (**b**) selected small molecule organics acting as adsorbates.

Four water-soluble anionic macrocyclic arenes, including WP5, WP6, WLT6, and WBpP6, bearing ten, twelve, eight, and eight carboxylate groups, respectively, were synthesized according to previously reported procedures (Figure 1a) [25,51,52,53]. All of the structures were fully characterized and confirmed using ^1^H NMR spectroscopy (Figures S1–S4), UV-vis absorption spectra (Figure S5), Fourier-transform infrared (FTIR) spectra (Figure S6), mass spectrometry (Figures S7–S10), and thermogravimetric analysis (Figure S11). To explore the liquid–liquid extraction performance of water-soluble pillararenes as adsorptive separation materials, we selected different types of low-molecular-weight organics with similar structures and close boiling points, such as short-chain haloalkanes, aromatics, and cyclic aliphatics, as adsorbates (Figure 1b). In addition, the effects of the pillararene concentration, proportion of mixtures, contacting time, and recycling time on separation performance were also carefully studied.

We first used a liquid–liquid extraction method to investigate the separation capability of WP5. In a typical procedure, the equimolar binary organics mixture was added to the aqueous solution of WP5. After being stirred, the resulting mixture was left to undergo extraction for a few minutes. Thereafter, the aqueous phase was separated from the organic phase and a simple extraction was performed using deuterated chloroform to extract the organics adsorbed by WP5 in the aqueous phase. The composition of the organic mixture adsorbed by the aqueous phase was fully characterized by ^1^H NMR spectra (Figures S12–S15). The detailed results are given below.

According to the data in Table 1, multi-substituted aromatics and cyclic aliphatics had greater separation efficiencies than short-chain haloalkanes. WP5 performed best in separating different types of organics analogs, followed closely by WP6. This could be because water-soluble pillararenes have hydrophobic cavities that can form a stable inclusion complex with aromatics. In contrast, the experimental results of WLT6 and WBpP6 were far from impressive, which may be because their cavities are too large to combine well with guest molecules.

The separation factor (α), which refers to the ratio of the relative content of the dominant extract before and after the extraction, was used to describe the extraction efficiency by different water-soluble pillararenes [54]. The relationship can be represented using the following equation, where *x_a_* and *x_b_* represent the mole quantity of components a and b in the mixed organic sources, and *y_a_* or *y_b_* is the mole quantity in the extracts:

$$\alpha =\frac{y_{b}/y_{a}}{x_{b}/x_{a}}$$

The primary variables in this experiment were the composition of organic mixtures, concentration of macrocyclic arenes, contact time, and recycling time. Different combinations have been tested to determine the optimal separation conditions. To the best of our knowledge, the adsorptive separation of liquid organics by WP6 has not been reported thus far. Therefore, we used WP6 and WP5 as examples in the following explorations to investigate the extraction efficiency for separating Tol/MCH mixtures in different conditions. We specifically chose these pillararenes because they previously displayed the best separation performance.

### 2.1. Effect of Pillararene Concentration and Stirring Time

**Figure 2:**
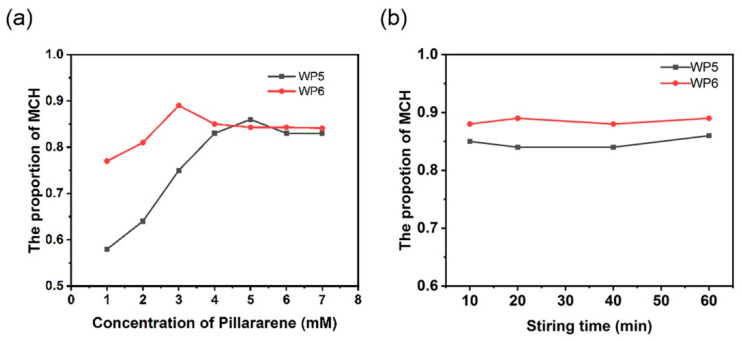
The proportion of MCH in the extraction phase at different (**a**) pillararene concentrations and (**b**) stirring times.

To gain more insight into the selectivity of pillararenes for mixtures separation, the effect of WP5 and WP6 concentrations on the proportion of MCH in the extraction phase was investigated. An equimolar Tol/MCH mixture (1:1) was prepared as the organic liquid source to be separated. It was found that the selectivity first increased, and then decreased slightly, as the pillararene concentration increased (Figure 2a). Due to the lower solubility of WP6 than WP5 in an aqueous solution, WP6 exhibited a sluggish selectivity for MCH at a low concentration range. When the separation efficiency reached the maximum value, the content of MCH in the aqueous phase underwent a slight decrease as the macrocycle concentration increased because the excess pillararenes could still adsorb a small amount of Tol. The best extraction efficiency (89%) was achieved when 3 mM WP6 was added in the extraction phase, and the optimal concentration of WP5 was 5 mM.

The optimal pillararene concentrations (3 mM for WP6 and 5 mM for WP5) were used to explore the effect of stirring time on the separation efficiency. The experimental results showed that increasing the stirring time to somewhere between 10 and 60 min does not change the final content level of MCH in the extraction phase (Figure 2b). This means that the specific complexation between water-soluble pillararenes and MCH proceeds rapidly and is easily completed within 10 min under an uncomplicated condition. Thus, we can conclude that host–guest interactions exist between pillararenes and MCH molecules. As contact time is an important criterion used to evaluate the efficiency of a separation technique, the fact that water-soluble pillararenes have such a short stirring time means that they can be feasibly used in a wide range of practical operations.

### 2.2. Effect of Single Component Content

**Figure 3:**
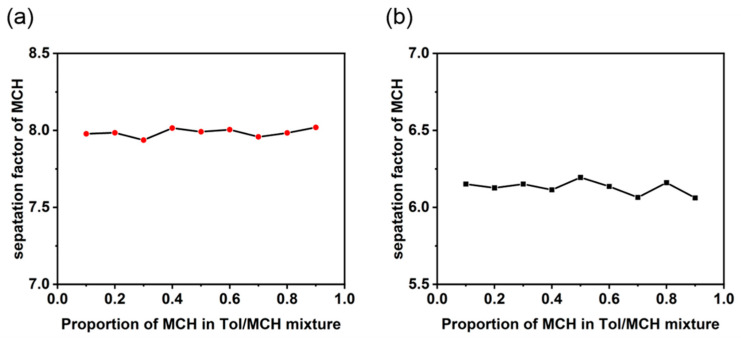
The relationship between the separation factor of MCH and the proportion of MCH after an extraction treatment by (**a**) WP5 and (**b**) WP6.

The extraction was subsequently performed using a different proportion of MCH in the Tol/MCH mixture. As the percentage of MCH was increased from 10% to 90%, there was no obvious change in the separation factor α of MCH after the organic phase was extracted by the arene aqueous solution (Figure 3). This result demonstrates that the pillararenes have excellent selectivity for MCH, and that the anionic macrocycles can be easily recycled from the mixed solution through the second round of liquid–liquid extraction by CDCl_3_. Meanwhile, MCH can be transferred into the organic phase of CDCl_3_. The average α value of WP6 is 8.08, which is slightly higher than that of WP5 (6.12), indicating that WP6 has better liquid–liquid phase extraction performance than MCH.

### 2.3. Curve of Extraction Equilibrium

Like the gas–liquid equilibrium curve, the extraction equilibrium curve describes the relationship between the composition of the Tol/MCH mixture sources and the extraction efficiency. Therefore, the extraction equilibrium curve is beneficial in practical applications. For a given starting mixture containing different percentages of MCH, the curve provides the proportion of MCH that can be obtained in the extraction phase. Moreover, the α value can also be calculated using this curve.

**Figure 4:**
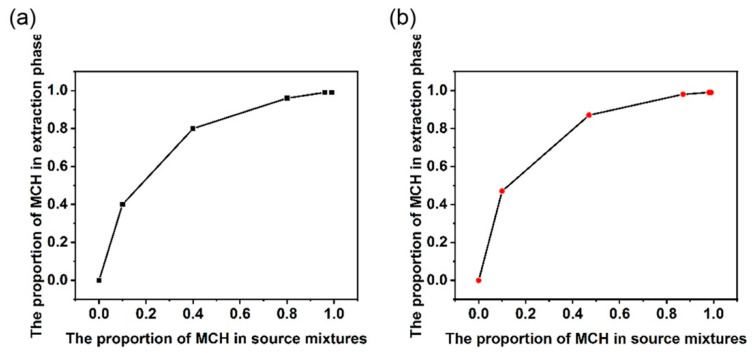
Extraction equilibrium curve of (**a**) WP5 and (**b**) WP6.

The extraction equilibrium curve in Figure 4 was obtained from a starting organic phase source of Tol/MCH (*v*:*v* = 1:9). The resulting extraction phase and MCH content were used directly in the next extraction cycle to obtain consecutive sample points in Figure 4. It was found that after three extraction cycles, the proportion of MCH increased from the initial 10% to 87% by WP5 solution and to 95% in the case of WP6. After five extraction cycles, an MCH content level of 99% could be achieved for both cases. Thus, the extraction equilibrium curve allows us to determine how many extraction cycles will be required to reach a specific separation target from a given starting mixture. This facilitates the practical application of water-soluble pillararenes in chemical separation and water purification.

### 2.4. Effect of Successive Cycles

**Figure 5:**
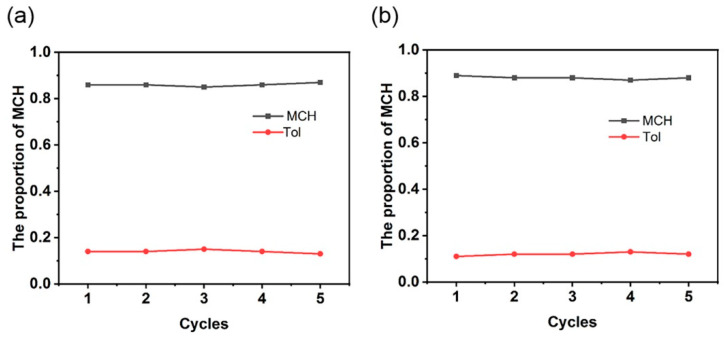
Relative uptakes of MCH and Tol by (**a**) WP5 and (**b**) WP6 for five cycles at 298 K.

Reusability is a critical factor in wastewater treatment, thus we attempted to reactivate the adsorbents WP5 and WP6 through washing, re-precipitation, and filtration. The recovered water-soluble anionic macrocycles were first sonicated in deionized water for 30 min at room temperature, then washed with DCM/water (1:1, *v*/*v*) and deionized water. Next, the aqueous solution of anionic macrocyclic arenes was evaporated and the precipitated macrocyclic salts were filtered and then desiccated at 90 °C in vacuo for 12 h to make sure that the absorbed guest molecules were completely released from the cavities. The recovered anionic pillararenes were reused for five successive cycles in a D_2_O solution containing Tol/MCH mixtures (1:1, *v*/*v*). No obvious deactivation was found, clearly demonstrating the stability of water-soluble pillararenes as adsorptive separating materials (Figure 5).

## 3. Materials and Methods

### 3.1. General Information

Starting reagents and solvents were commercially available and used as received unless stated otherwise. Deionized water used in the relevant experiments was purified using an experimental water system (Lab-UV-20). All four macrocyclic arenes, including WP5, WP6, WLT6, and WBpP6, were synthesized according to the published procedures [25,51]. UV-vis spectra were recorded on a Shimadzu UV-2550 instrument. Fourier transform infrared spectroscopy (FT-IR) spectra were recorded on a Vertex 80 V spectrometer. Mass spectra were recorded on a Bruker Agilent1290-micrOTOF Q II High-resolution (HR) mass spectrometry instrument. Thermogravimetric analysis was performed by using a NETZSCH STA 449F3 QMS403D\Bruker V70 instrument, and each sample was heated to a temperature of 600 °C at a rate of 10 °C min^−1^ under a nitrogen atmosphere. ^1^H NMR spectra were recorded on a Bruker Avance III 400 MHz spectrometer at room temperature. Solution ^1^H NMR spectra were recorded using the deuterated solvent as the lock and the residual solvent as the internal reference.

### 3.2. Liquid-Liquid Extraction Experiments

WP6 (250 mg) was dissolved in 50 mL neat water to get a 3.0 mM WP6 aqueous solution. Then, 50 mL Tol/MCH mixtures (1:1, *v*/*v*) were added into the above solution. After stirring for 30 min, the water phase was separated from the benzene isomers phase. The emulsifying benzene isomers, which were liquid in the water phase, could be removed by centrifugation and filtration. The water phase was extracted by an organic solvent, CDCl_3_, for the ^1^H NMR experiment [55].

## 4. Conclusions

In conclusion, we demonstrated that water-soluble pillararenes could be used as rational separation adsorbents for low-molecular-weight organics. The carboxylate groups in the backbones of macrocycles provide them with good water solubility and the presence of hydrophobic intrinsic cavities allow them to selectively adsorb and separate specific organics. Although all of the pillararenes considered have a certain degree of adsorption capacity, the size of the cavity is the main factor that determines the separation performance. Although there have been previous reports of using pillar[6]arene to separate organic matters in the solid crystal state, the current work is the first time that water-soluble WP6 was used to separate and purify organics in the aqueous phase by liquid–liquid extraction. This not only expands the application scope of pillar[6]arene, but also provides a reference value for the application of other macrocyclic arenes in the future. In addition, the water-soluble anionic pillararenes not only have good separation properties, but also exhibit excellent stability and cycling performance. Therefore, this work creates more possibilities for the construction of high-performance adsorbents in organics separation and water purification, which is of great significance for energy conservation and environmental protection.

## Figures and Tables

**Table 1:** Composition of extracts after extraction by four water-soluble pillararenes according to the results of ^1^H NMR spectra.

Mixtures	WP5	WP6	WLT6	WBpP6
OX	0.15	0.44	/	/
MX	0.85	0.56		
MCT	0.96	0.75	0.46	0.53
OCT	0.04	0.25	0.54	0.47
MBT	0.93	0.73	0.59	0.44
OBT	0.07	0.27	0.41	0.56
St	0.36	0.54	0.64	/
EB	0.64	0.46	0.36	
Tol	0.14	0.11	/	/
MCH	0.86	0.89		
Br-n-bu	0.76	0.48	0.49	0.75
Br-i-bu	0.14	0.23	0.27	/
Br-s-bu	0.1	0.29	0.24	0.25
Br-n-Pr	0.71	0.43	0.52	0.55
Br-i-Pr	0.29	0.57	0.48	0.45
Cl-n-bu	0.60	0.42	0.48	0.47
Cl-i-bu	0.40	0.57	0.52	0.53

A lack of data means that there were no organics in the water phase to be extracted. Relevant results were obtained from the average of three independent extraction/release experiments.

## Data Availability

Not applicable.

## Supplementary Materials

The following supporting information can be downloaded at: https://www.mdpi.com/article/10.3390/molecules27238554/s1, Figures S1–S11: Characterization of four water-soluble anionic pillararenes; Figures S12–S15: 1H NMR spectra of the extraction phase.

## Sample Availability

Samples of the compounds are available from the authors.
