# Supplementary material for: Separation of Low-Molecular-Weight Organics by Water-Soluble Macrocyclic Arenes
Source: Molecules. 2022 Dec 5;27(23):8554. doi: 10.3390/molecules27238554 (PMC9736317; doi:10.3390/molecules27238554)
Supplement: Supplementary file 1 [file molecules-27-08554-s001.zip › molecules-2059302-supplementary.pdf]

Supplementary Materials

Separation of Low-Molecular-Weight Organics by Water-Soluble Macrocyclic Arenes

Wenhui Wang, Zheng Li, Chunli Song, Jie Yang and Yingwei Yang*

International Joint Research Laboratory of Nano-Micro Architecture Chemistry, College of Chemistry, Jilin University, 2699 Qianjin Street, Changchun 130012, China; wangwh22@mails.jlu.edu.cn (W.W.); lz20@mails.jlu.edu.cn (Z.L.); songcl20@mails.jlu.edu.cn (C.S.); jieyang@jlu.edu.cn (J.Y.)

* Correspondence: ywyang@jlu.edu.cn

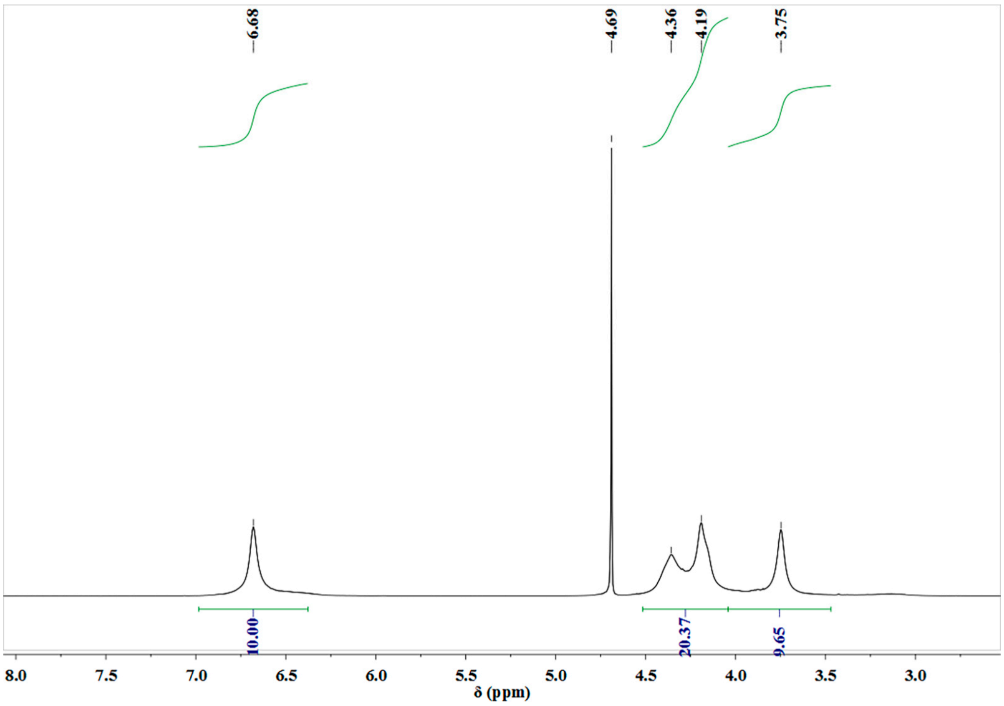

Figure S1. ^{1}H NMR spectrum (400 MHz, 298 K, D_2O) of **WP5**.

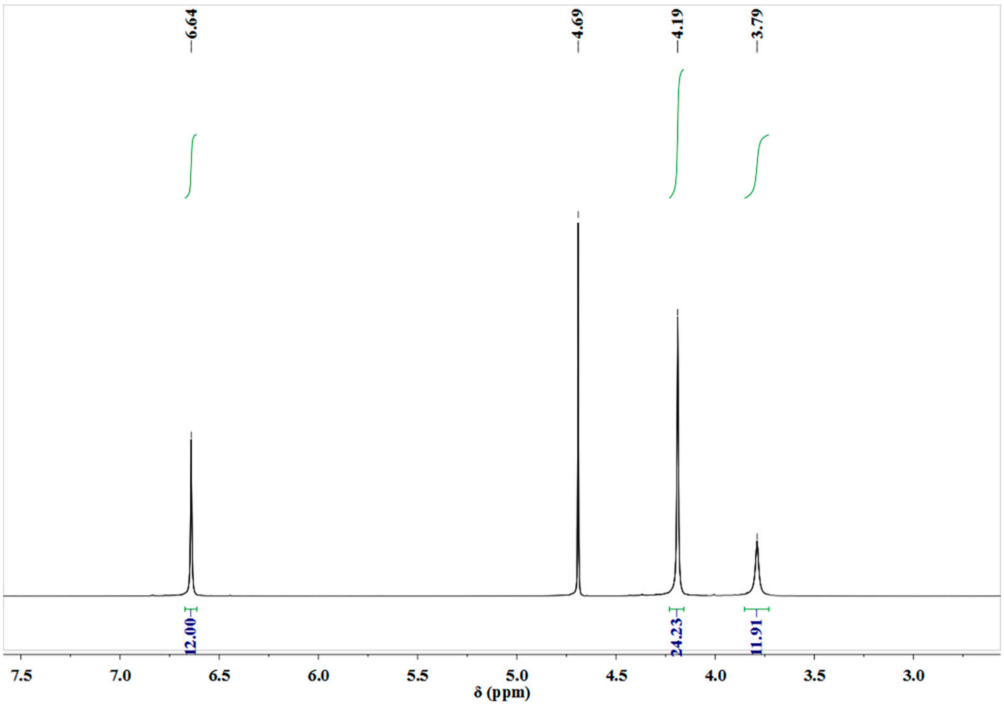

Figure S2. ^{1}H NMR spectrum (400 MHz, 298 K, D_2O) of **WP6**.

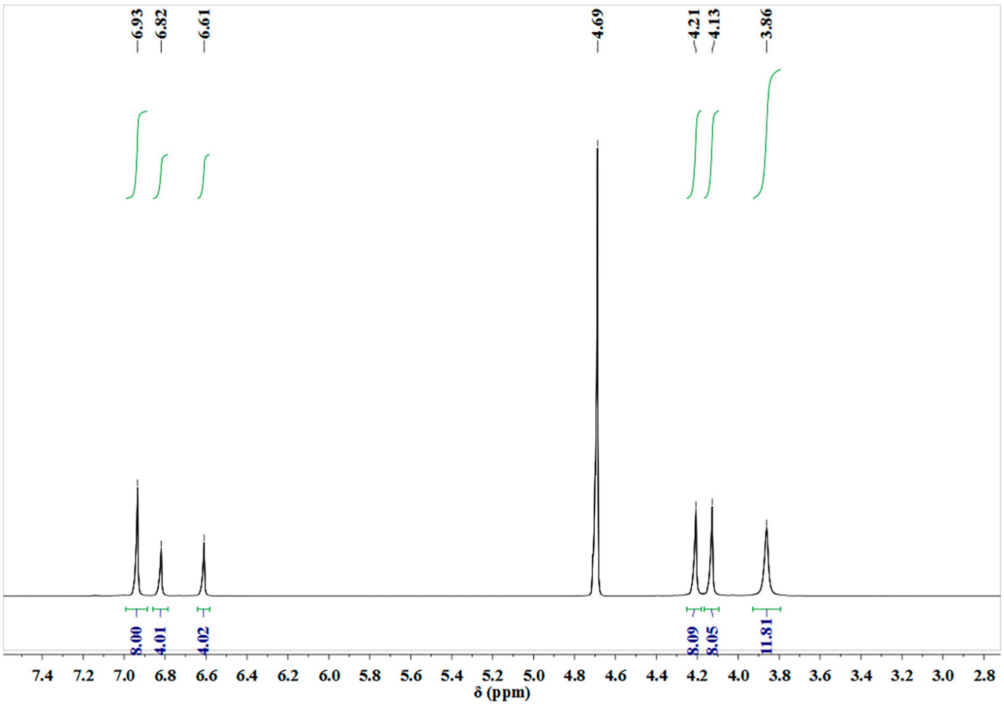

Figure S3. ^{1}H NMR spectrum (400 MHz, 298 K, D_2O) of **WLT6**.

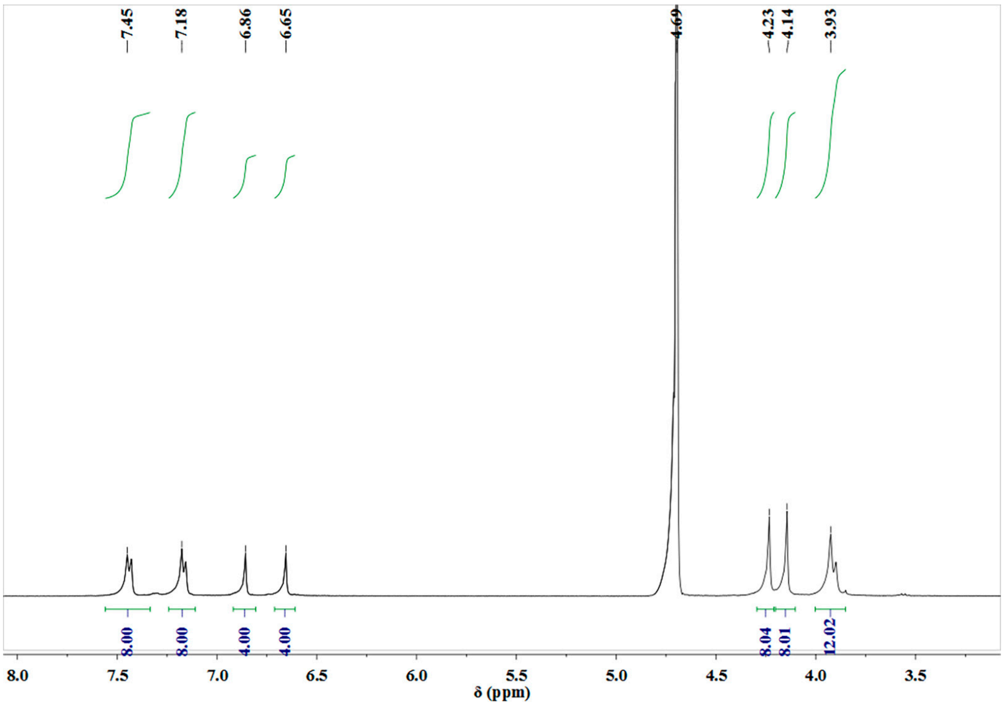

Figure S4. ^{1}H NMR spectrum (400 MHz, 298 K, D_2O) of **WBpP6**.

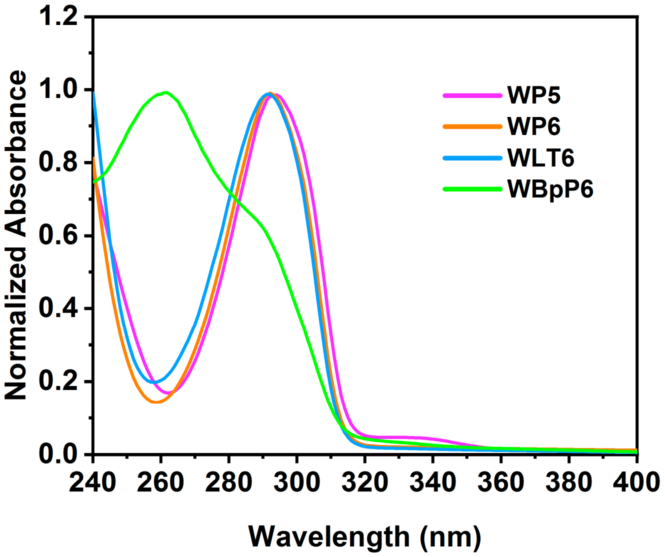

Figure S5. Normalized UV-vis absorption spectra of **WP5**, **WP6**, **WLT6** and **WBpP6** in deionized water (concentration, 10^{-6} M).

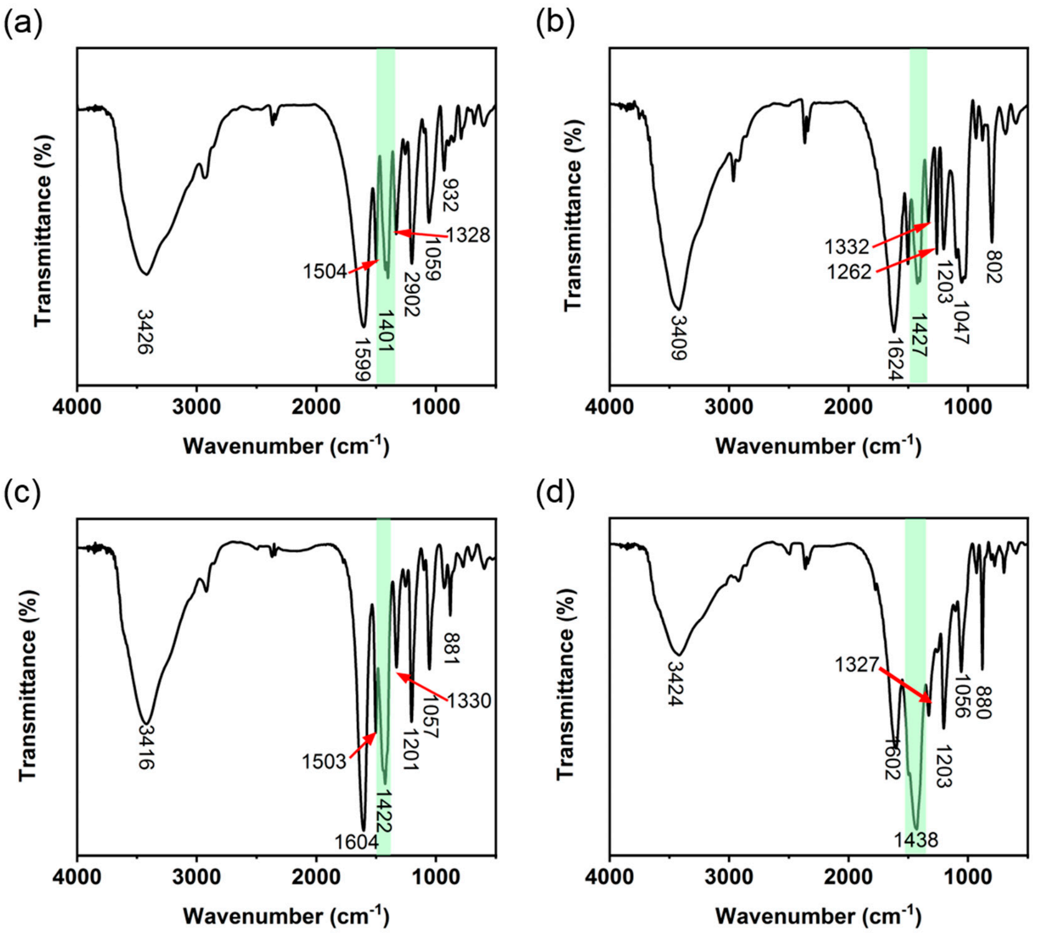

Figure S6. Fourier-transform infrared (FTIR) spectra of (a) **WP5**, (b) **WP6**, (c) **WLT6** and (d) **WBpP6** and the characteristic peaks of -COO⁻ groups have been highlighted.

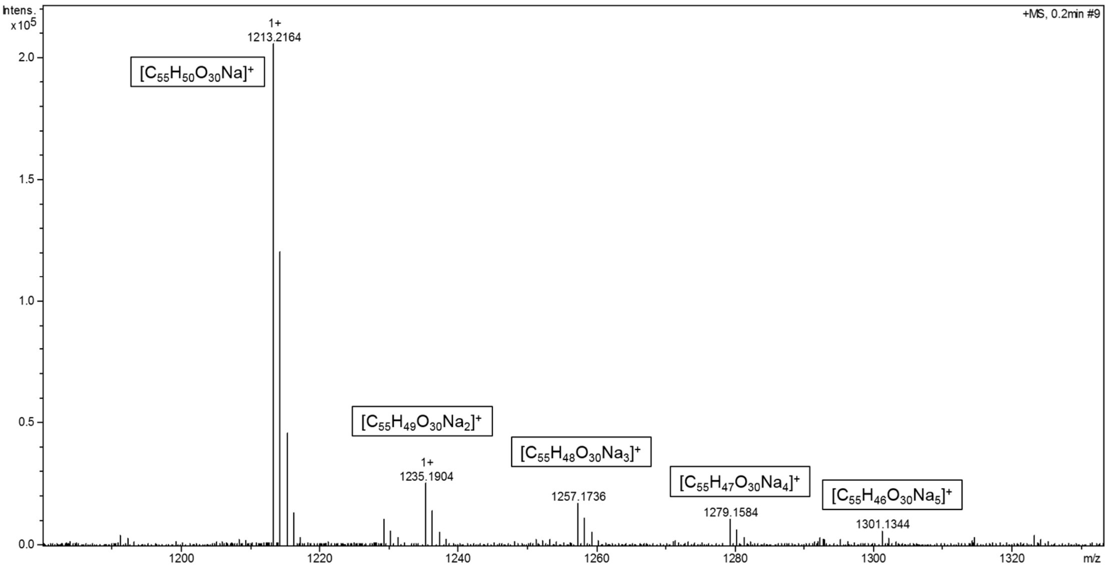

Figure S7. Electrospray ionization mass spectrometry of **WP5**. Assignment of the main peak: m/z 1213.2279 [M-9Na+10H]$^+$.

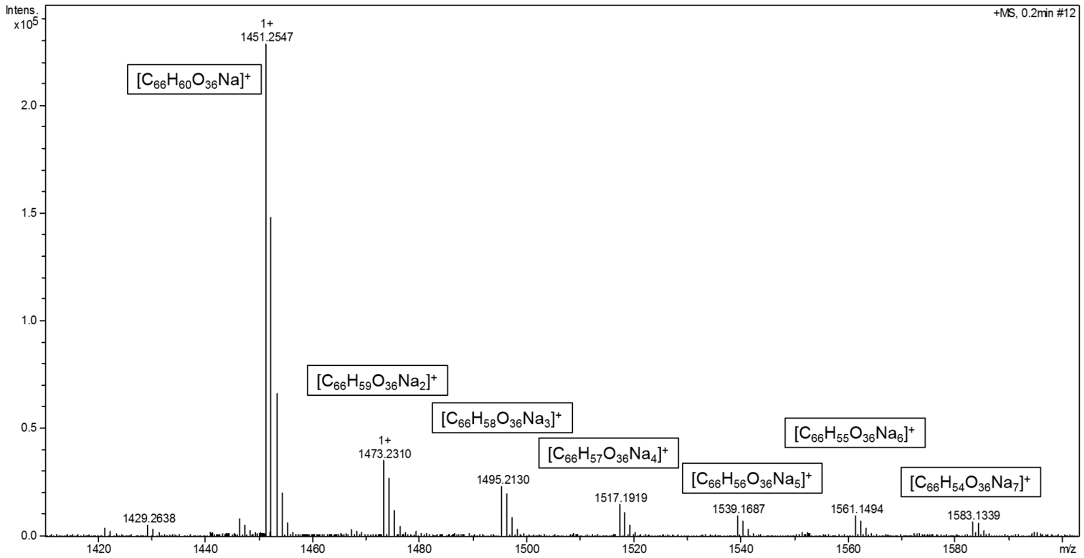

Figure S8. Electrospray ionization mass spectrometry of **WP6**. Assignment of the main peak: m/z 1451.2756 [M-11Na+12H]$^+$.

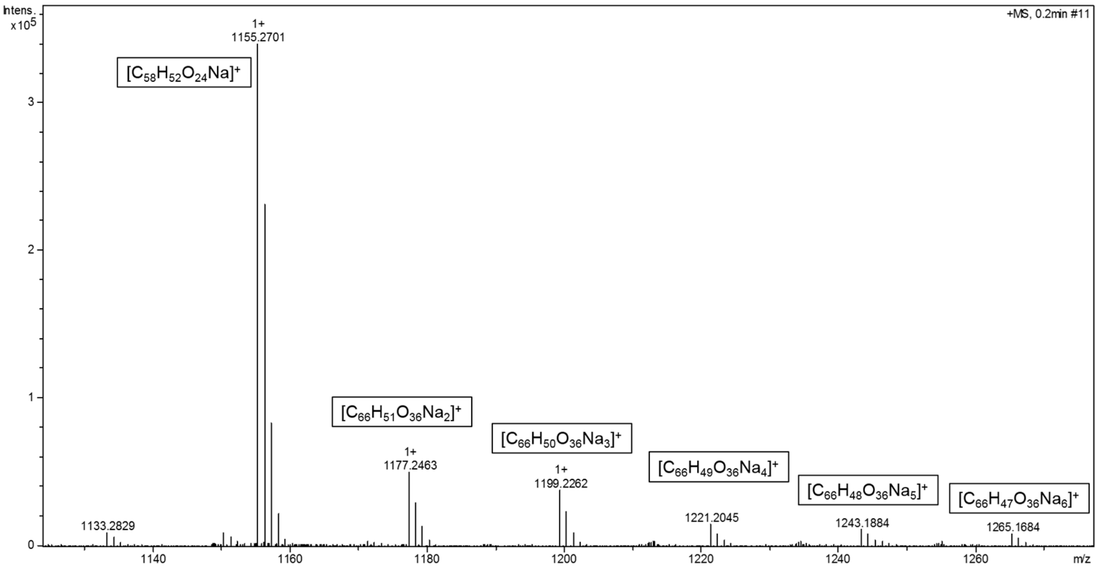

Figure S9. Electrospray ionization mass spectrometry of W**LT6**. Assignment of the main peak: m/z 1155.2741 [M-7Na+8H]$^+$.

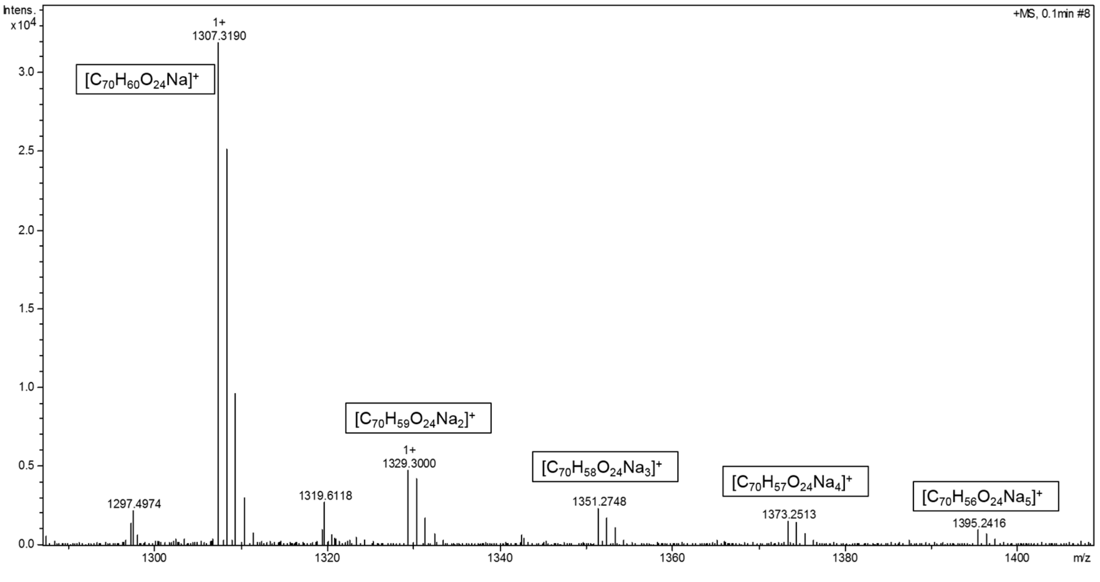

Figure S10. Electrospray ionization mass spectrometry of **WBpP6**. Assignment of the main peak: m/z 1307.3367 [M-7Na+8H]$^+$.

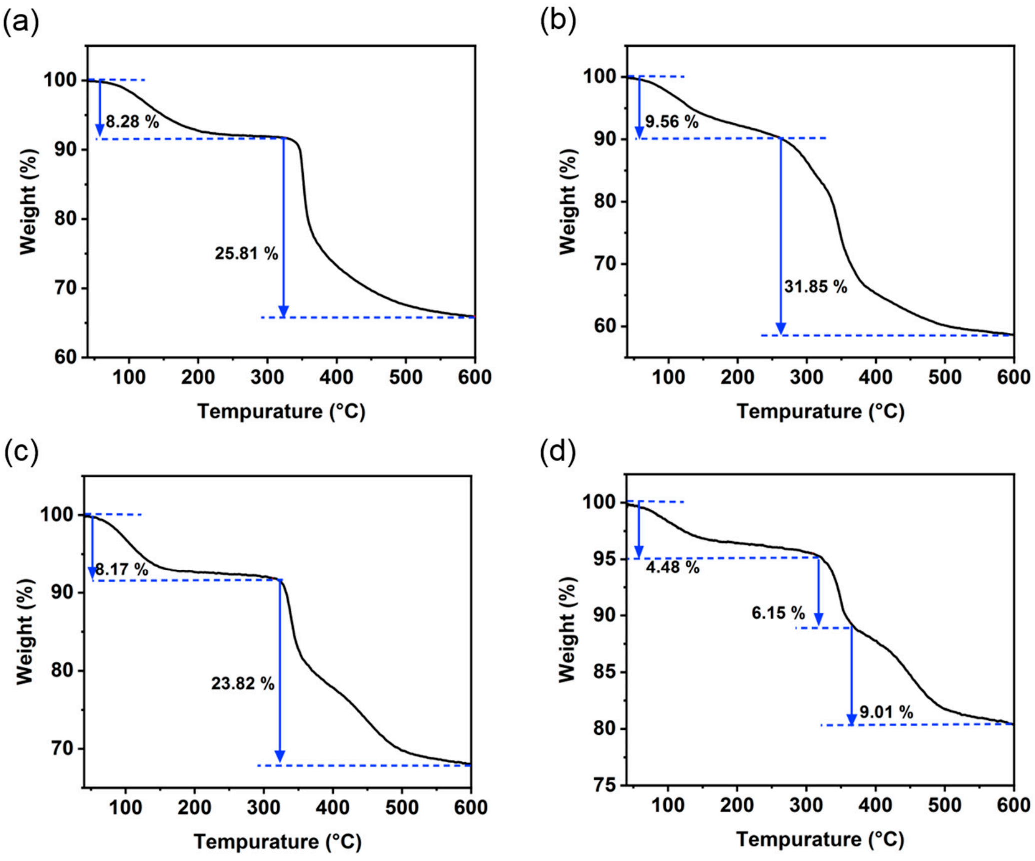

Figure S11. Thermogravimetric analysis of (a) **WP5**, (b) **WP6**, (c) **WLT6** and (d) **WBpP6**.

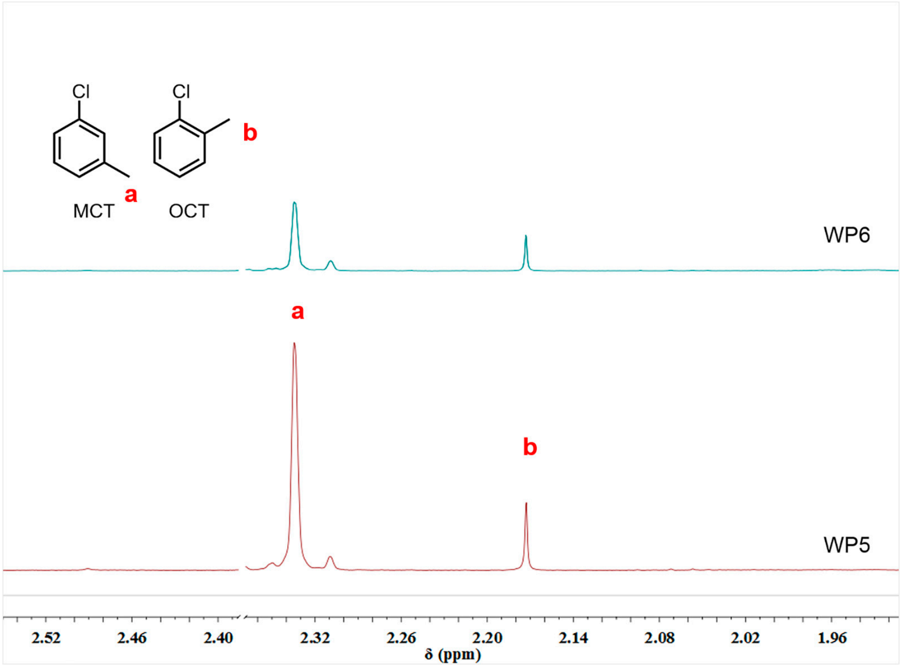

Figure S12. Partial ^1H NMR spectra (400 MHz, $CDCl_3$, 298 K) of extracted organic phase from MCT/OCT mixture by **WP5** and **WP6**.

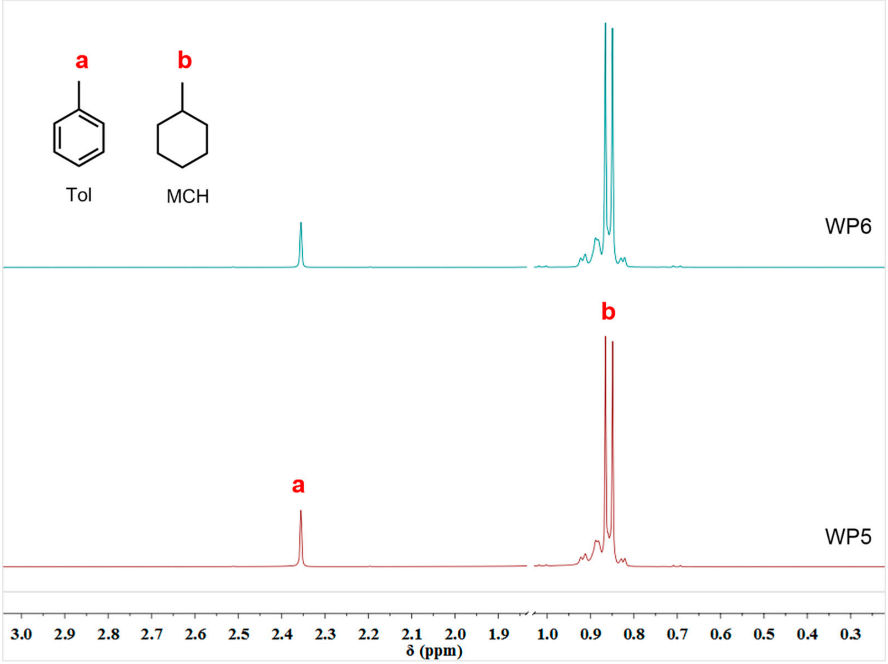

Figure S13. Partial ^1H NMR spectra (400 MHz, $CDCl_3$, 298 K) of extracted organic phase from Tol/MCH mixture by **WP5** and **WP6**.

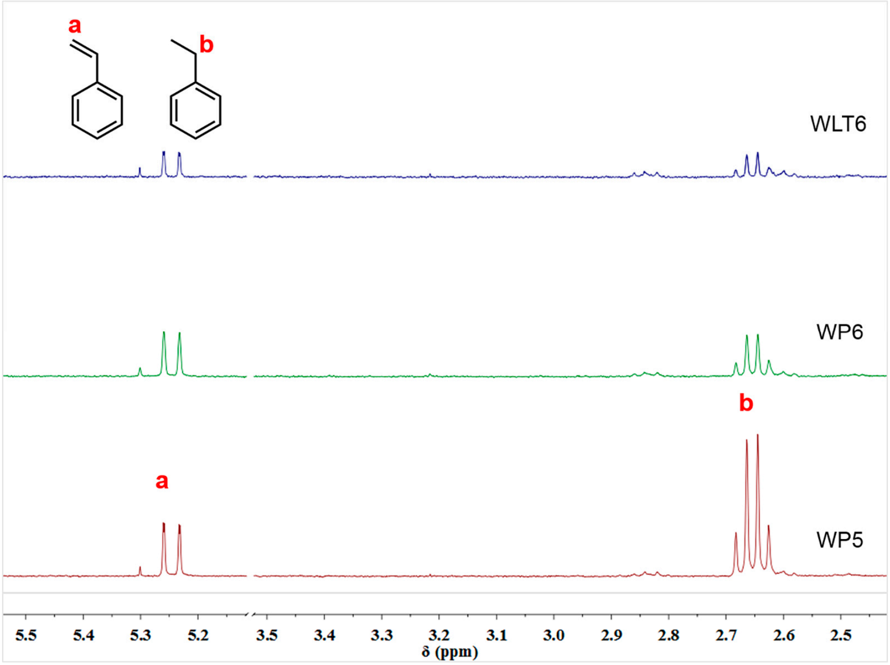

Figure S14. Partial ^{1}H NMR spectra (400 MHz, $CDCl_3$, 298 K) of extracted organic phase from St/EB mixture by **WP5**, **WP6** and **WLT6**.

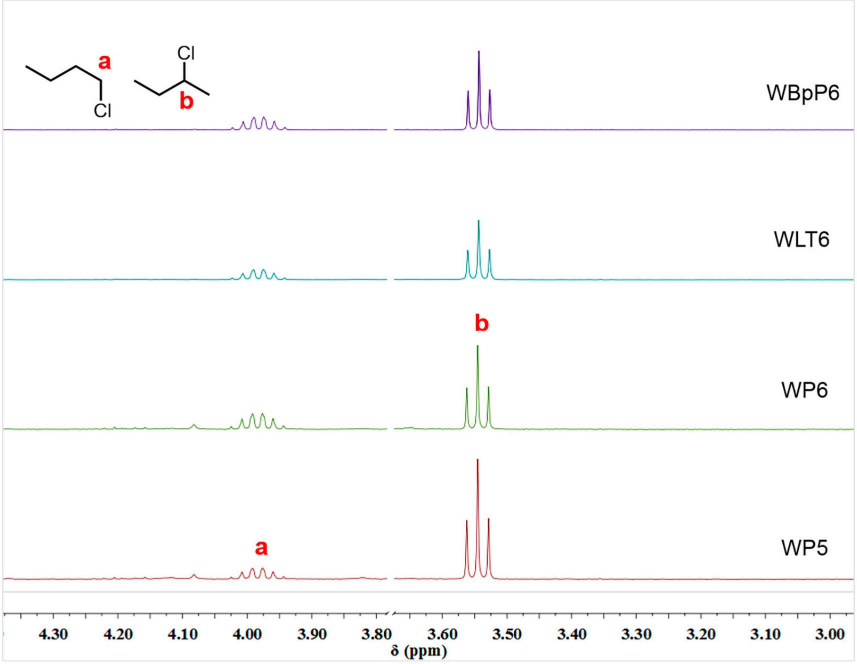

Figure S15. Partial ^{1}H NMR spectra (400 MHz, $CDCl_3$, 298 K) of extracted organic phase from Cl-n-bu/Cl-i-bu mixture by **WP5**, **WP6**, **WLT6** and **WBpP6**.
